# A robot-aided visuo-motor training that improves proprioception and spatial accuracy of untrained movement

**DOI:** 10.1038/s41598-017-16704-8

**Published:** 2017-12-06

**Authors:** Naveen Elangovan, Leonardo Cappello, Lorenzo Masia, Joshua Aman, Jürgen Konczak

**Affiliations:** 1 Human Sensorimotor Control Laboratory, School of Kinesiology, University of Minnesota, Minneapolis, MN USA; 2 Center for Clinical Movement Science, University of Minnesota, Minneapolis, MN USA; 3 School of Engineering and Applied Sciences, Harvard University, Cambridge, MA USA; 4 Wyss Institute for Biologically Inspired Engineering at Harvard University, Boston, MA USA; 5 School of Mechanical and Aerospace Engineering, Nanyang Technological University, Singapore, Singapore; 6 Neuromodulation Research Center, Department of Neurology, University of Minnesota, Minneapolis, MN USA

## Abstract

Proprioceptive function can become enhanced during motor learning. Yet, we have incomplete knowledge to what extent proprioceptive function is trainable and how a training that enhances proprioception may influence performance in untrained motor skills. To address this knowledge gap, healthy young adults (N = 14) trained in a visuomotor task that required learners to make increasingly accurate wrist movements. Using a robotic exoskeleton coupled with a virtual visual environment, participants tilted a virtual table through continuous wrist flexion/extension movements with the goal to position a rolling ball on table into a target. With learning progress, the level of difficulty increased by altering the virtual ball mechanics and the gain between joint movement and ball velocity. Before and after training, wrist position sense acuity and spatial movement accuracy in an untrained, discrete wrist-pointing task was assessed using the same robot. All participants showed evidence of proprioceptive-motor learning. Mean position sense discrimination threshold improved by 34%. Wrist movement accuracy in the untrained pointing task improved by 27% in 13/14 participants. This demonstrates that a short sensorimotor training challenging proprioception can a) effectively enhance proprioceptive acuity and b) improve the accuracy of untrained movement. These findings provide a scientific basis for applying such somatosensory-based motor training to clinical populations with known proprioceptive dysfunction to enhance sensorimotor performance.

## Introduction

It is well established that proprioceptive afferent signals from mechanoreceptors such as muscle spindles or Golgi tendon organs are essential for the control of muscle tone, the control of posture, and for the conscious awareness of body and limbs^1^. It is further known that altered proprioception due to deafferentation severely affects both spatial^2^ and temporal aspects of movement^3^. Moreover, numerous neurological conditions such as Parkinson’s disease^4–7^, stroke^8–10^, and peripheral neuropathy^11^ cause not only motor deficits, but are also associated with proprioceptive impairment. While the functional connection between proprioception and motor control has long been recognized, we have only an incomplete understanding to what extent the proprioceptive sense is trainable and to what extent improvements in proprioceptive function translate to improvements in motor function.

The notion that proprioception can be trained and that improved proprioceptive function may either yield superior motor performance or aid motor recovery, has been promoted for the coaching of sport skills, in rehabilitation medicine and physical therapy. In this context, the term *proprioceptive training* describes a wide array of interventions aimed at improving proprioceptive function with the ultimate goal to improve motor performance^12^. Proprioceptive training interventions may include active movement/balance exercises^13–15^, passive movement training^16–18^, somatosensory stimulation training^19–21^, somatosensory discrimination training^22–24^, or multi-modal sensory systems training^25^. Frequently, studies on proprioceptive training reported motor measures like Berg Balance scale scores^26^, center of pressure velocity^27^, and timed-up-and-go movement times^28^ to demonstrate behavioral improvements believed to be a result of improved proprioceptive function. Unfortunately, few investigations actually reported proprioceptive outcome measures to demonstrate the effectiveness of a proprioception-based training approach. The results of those studies are mixed. Some studies employing active movement exercises with visual feedback showed substantial improvements in proprioceptive function^16,18,20^. In contrast, other studies reported gains in motor, but not in proprioceptive function^29^ (patients with ACL reconstruction after neuromuscular training), or neither sensory nor motor improvement^28^ (patients with Parkinson’s disease after low-frequency whole-body vibration). Unfortunately, the heterogeneity of the reported proprioceptive training interventions and outcome measures have made it difficult to reach definitive conclusions on the usefulness of this approach for motor skill learning and rehabilitation.

What is missing, at present, are investigations that systematically evaluate the possible magnitude of changes in proprioceptive accuracy that can be achieved with sensorimotor training and to determine, if and how such proprioceptive changes transfer to motor function. Specifically, it would be meaningful to understand, if observable improvements in motor function are task- or movement specific, or if they transfer to untrained motor behaviors. That is, is such training highly specific or are signs of enhanced performance observable in movements that were not part of training? Therefore, the purpose of this study was a) to examine the magnitude of change in proprioceptive acuity that can occur with a visuomotor-based training that challenges proprioception and fine motor control, and b) to determine if such changes in sensory precision transfer to general movement accuracy. Healthy, neurologically normal adults used a robotic exoskeleton to train goal-directed wrist movements. We employed an active-movement-based training with visual feedback, because such training had shown to produce the measurable gains in proprioceptive function in earlier studies (for a review see^12^). Before and after training, we obtained wrist joint position sense discrimination thresholds as measures of proprioceptive learning and the spatial movement accuracy error during previously untrained, discrete wrist pointing movements as a measure of motor transfer.

## Results

### Learning-related changes in proprioceptive discrimination thresholds

**Figure 1:**
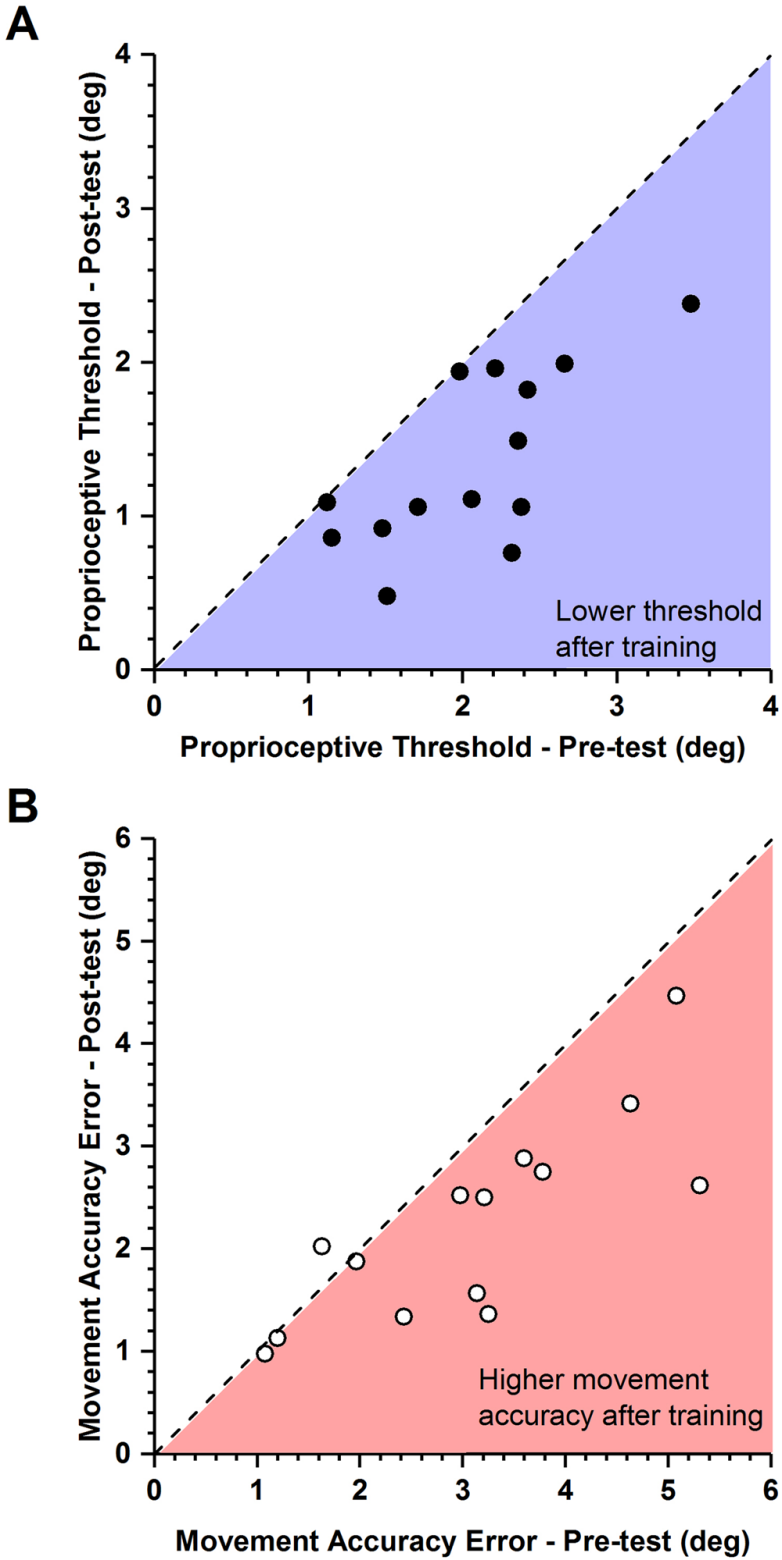
Effects of training on proprioceptive thresholds and movement accuracy. Each data point indicates the pre-post training values of a single participant. (**A**) Proprioceptive discrimination thresholds before and after training. The dashed line indicates the line of equality, indicating no change as a function of training. Note that all thresholds were either on or below the line of equality. Blue area marks the region of lower thresholds after training. (**B**) Movement accuracy error before and after training. Light red area marks the region of higher movement accuracy (lower error) after training. Note that that 10/14 participants exhibited movement accuracy error values within that region.

**Figure 2:**
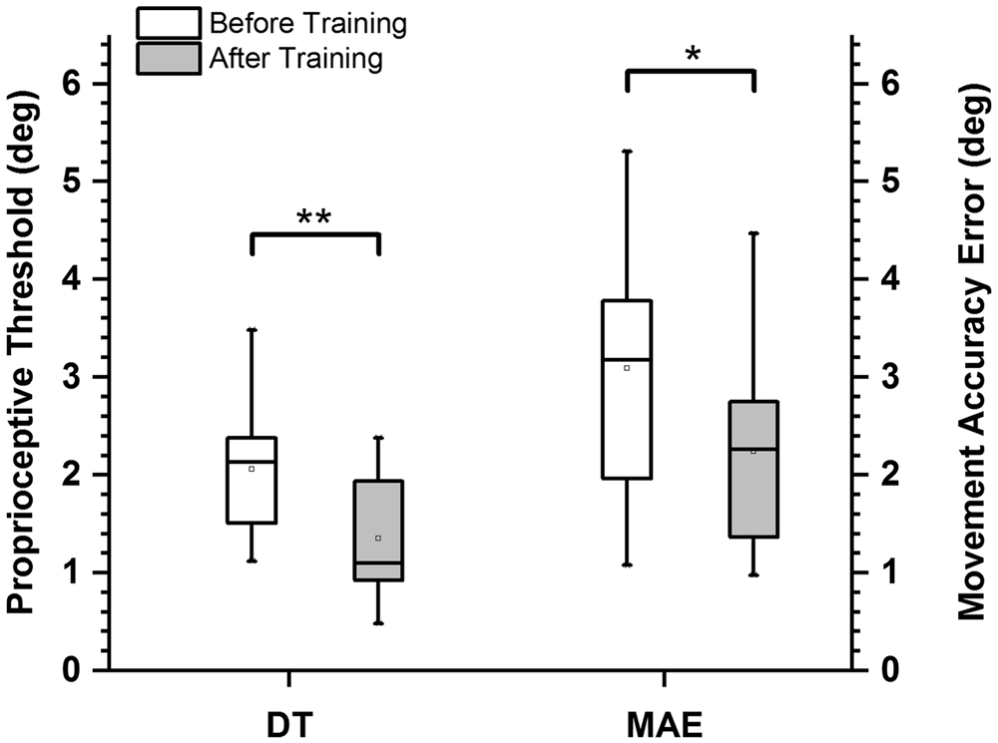
Mean effects of training on proprioceptive discrimination thresholds and movement accuracy error. **Indicates p < 0.005 and * indicates p < 0.01. DT = discrimination threshold, MAE = movement accuracy error.

At the end of training, all 14 participants had either maintained or reduced their proprioceptive discrimination thresholds (see Fig. 1A for individual participant data). In order to determine the magnitude of the overall learning effect on proprioceptive acuity, we computed the mean proprioceptive discrimination threshold (DT) across all participants (before training: mean ± SD: 2.05° ± 0.61°; after training: 1.38° ± 0.56°). A respective one-way repeated measures ANOVA on DT revealed that the difference in DT before and after training was statistically significant (F_1,13_ = 11.51; p = 0.005), indicating that the applied training-induced improvements in proprioceptive acuity (see Fig. 2 for population data). In relative terms, the training related improvement in the proprioceptive discrimination thresholds ranged from 2% to 68% with an average improvement of 34% across all participants.

### Learning-related changes in movement accuracy

At the end of training, 13 of the 14 participants had either maintained or decreased their movement accuracy error (MAE). One participant showed an increase in movement accuracy error after training (see Fig. 1B for individual participant data). Mean movement accuracy error across all participants was 3.09° ± 1.34° SD before training and 2.24° ± 0.98° SD after training. To determine the learning-induced changes in movement accuracy error, a pre-post training one-way repeated measures ANOVA on MAE was performed, yielding a significant reduction in movement accuracy error after training (F_1,13_ = 11.9; p = 0.005). These results indicated that, on average, movement accuracy error in the untrained discrete motor task had significantly decreased in magnitude (see Fig. 2 for the population data). The relative improvement in the movement accuracy error ranged from 5% to 51% with an average improvement of 27% across all participants.

### Relationship between initial performance and the magnitude of learning

To understand, how initial sensory or motor performance affected the extent of possible learning, a Pearson’s product-moment correlation analysis between the pre-test sensory and motor function measures and their respective absolute improvements with training was performed. Correlating the proprioceptive discrimination thresholds before training and the absolute reduction in proprioceptive discrimination thresholds after training yielded a significant correlation of r = 0.49 (p < 0.05). Similarly, movement accuracy error before training correlated significantly with the absolute decrement in movement accuracy error after training with r = 0.69 (p < 0.002). Those participants who had lower sensory acuity or larger errors in movement accuracy prior to training, tended to exhibit larger degrees of learning, when compared to those with high proprioceptive acuity and/or movement accuracy.

### Relationship between changes in sensory and motor performance

**Figure 3:**
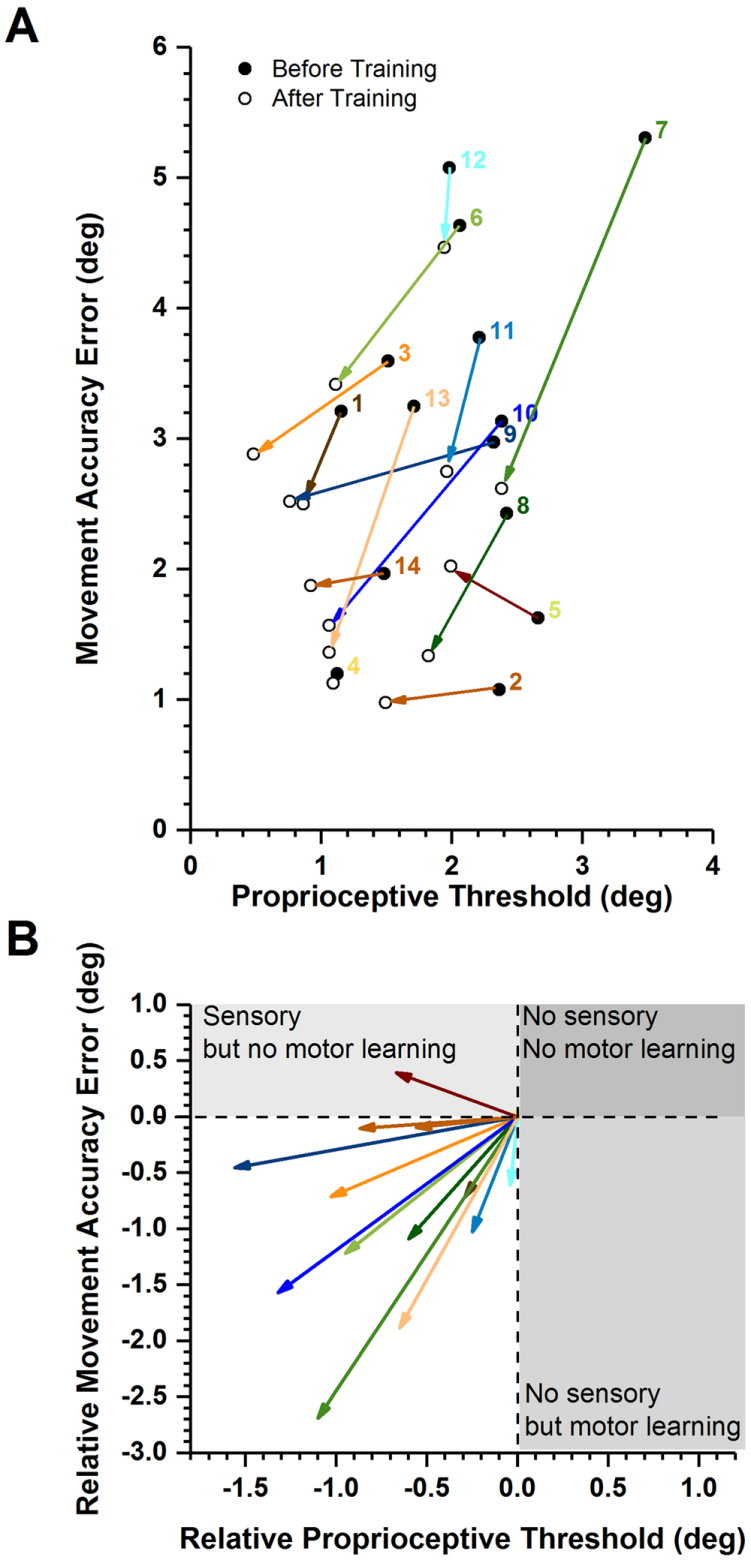
Mapping gains in proprioceptive acuity against gains in movement accuracy. (**A**) Each participant’s sensorimotor gain is depicted as a vector. The coordinates of proprioceptive discrimination threshold and movement accuracy error score before training represent the origin of the vector. The vector length indicates the magnitude and the angle the direction of change. (**B**) Vectorgram of the relative gain. The same vectors as in (**A**) are shown with their origins aligned to coordinates (0,0). Vector length indicates relative change. Note that all but one vectors fall into quadrant of concurrent sensory and motor learning.

We further examined to what extent training induced improvements in somatosensory function related to changes in motor performance. Figure 3 maps the absolute change in movement accuracy as a function of the training induced changes in proprioceptive acuity. Concurrent changes in sensory and motor performance were observed in 13 out of 14 participants (86%). That is, both proprioceptive and motor learning occurred together with training. However, a large improvement in sensory acuity did not uniformly translate into a large improvement in movement accuracy. To understand the training-induced differences, each participant’s sensory and motor performance was represented as a vector of their proprioceptive threshold and movement accuracy error (Fig. 3b). The respective mean vector angle across participants was 47.1° indicating that on average all participants improve similarly in proprioceptive function and movement accuracy (Rayleigh z_10.18_, p < 0.001).

## Discussion

This study addressed the trainability of the joint position sense. It sought to achieve two aims: First, to examine the magnitude of change in proprioceptive acuity after completing a sensorimotor training that challenges proprioception. Second, to determine if and to what extent changes in proprioceptive acuity transfer to general movement accuracy. Our main results show that a brief robot-assisted sensorimotor training improves proprioceptive acuity. Moreover, it documents that these sensory changes are accompanied by a concurrent improvement in movement accuracy of the same joint. This enhanced movement accuracy was visible in movements that were not part of the training.

### Gains in proprioceptive function

Our brief sensorimotor training challenged the ability to sense small differences in joint position as it required participants to make continuous, small-amplitude wrist movements. We found that the training induced fast, measurable changes in proprioceptive thresholds in the majority of participants (see Fig. 1A). To appreciate the magnitude of proprioceptive learning, consider that human adults reliably detect joint position changes as small as 1–2° and joint motion as low 1°/s^5,30–32^. In this study, the mean discrimination threshold before training was 2.05° with respect to the 15° reference position. At the end of training, the mean threshold had dropped to 1.38° (see Fig. 2). That is, on average, the group of healthy adults showed a 34% reduction in their discrimination thresholds. This magnitude of change is in line with previous work that evaluated changes in proprioceptive function after training^14,15,29,33,34^. A recent systematic review reported improvements in proprioceptive function ranging between 18% to 58% for variables such as active joint matching position error or passive motion thresholds^12^.

Because the exact measurement of proprioceptive function is complex, numerous studies resorted to report motor measures like Berg Balance scale scores^26^, center of pressure velocity^27^, and timed-up-and-go movement times^28^ as indirect measures of proprioceptive function. Here, we quantified proprioceptive acuity using a psychophysical threshold paradigm – an established method considered to be a gold-standard in measuring sensory acuity^35^. The method has shown to generate highly sensitive and reliable measures of proprioceptive function^30,36^. Because it uses passive joint movement controlled by our robotic device, the contribution of somatosensory afferent signals was isolated from processes of sensorimotor integration or motor control. Both these processes are necessarily present during active volitional movement and are known to affect proprioceptive acuity. Consequently, we can be reasonably certain that the observed gains in proprioceptive acuity represent the result of somatosensory learning and are not the result of improved underlying motor control or motor learning processes.

While this study examined changes in proprioceptive acuity during motor skill learning, other studies evaluated changes in proprioception as a function of adaptive sensorimotor learning. One experimental approach is to misalign visual information of a limb or target during motor learning and then to evaluate the recalibration of proprioceptive system with respect to the visual system after training. These studies show that proprioceptive recalibration can be incomplete and is slower than motor adaptation, which indicates that likely two separate neural processes underlie sensory recalibration and adaptive motor learning^37–39^. In contrast, in the current study neither visual nor proprioceptive information was altered. Consequently, no bi- or multimodal sensory recalibration or intersensory remapping was necessary to succeed in the task. This implies that the observed gains in proprioceptive acuity are not explained by processes of proprioceptive recalibration or adaptive sensorimotor learning. We rather observed a concurrent change in proprioceptive and motor skill learning measures, which likely reflect processes of short-term neural plasticity in somatosensory and motor cortical areas (for a review see^40^).

### Improvements in untrained motor function

Recent neurophysiological research on the functional link between proprioceptive and motor learning demonstrated that this relationship is bidirectional^40^. That is to say, proprioceptive function may be enhanced after learning a motor task^34^ or, vice versa, proprioceptive sensory training may improve motor learning^41^. For example, after people learned to discriminate different vibrations applied to a thumb muscle, their rate of learning in a thumb abduction task using the same muscle increased^42^.

Here, we tested, if a measurable change in sensory acuity during sensorimotor learning translated into improved movement accuracy in a novel motor task. We found that the movement accuracy error decreased in the majority of participants (13/14) after training. Concurrent gains in proprioceptive acuity and movement accuracy were observed in all but one participant (see Fig. 3). The overall magnitude of the gains in proprioceptive acuity (+34%) and movement accuracy (+27%) were comparable (see Fig. 2).

In order to understand the implications of this result, consider that this improvement in spatial movement accuracy is not the direct result of motor learning, because the movements of the transfer task were not practiced. Participants had trained to make continuous, small amplitude movements using vision, while the motor transfer task required them to make discrete, goal-directed movements without vision. This finding is consistent with the notion that gains in sensory function transferred, i.e. they helped to improve motor function. Specifically, gains in proprioceptive acuity translated into improved spatial movement accuracy. However, one needs to be mindful that the transfer task involved the same joint and the same joint degree of freedom (flexion/extension). That is, we have evidence of a limited or local transfer. In other words, this sensorimotor transfer is joint-specific, or more precisely, specific to the trained joint degree of freedom. To fully understand the *specificity of training* issue, one would have to design transfer tasks to other degrees of freedom of the trained joint and to test proprioceptive function and motor performance in adjacent joints.

### The neuroanatomical basis for a transfer of sensory learning to motor function

The afferent pathways that link peripheral mechanoreceptors with spinal cord, cerebellum and sensorimotor cortex are well known^43^. In addition, there are extensive reciprocal connections between somatosensory and motor cortical areas that link somatosensory and parietal cortices with motor, premotor and prefrontal cortex. Primary and premotor cortex have somatosensory receptive fields^44,45^ and neurons in ventral premotor cortex and supplementary motor area (SMA) are involved in the transformation of sensory information into motor actions^46^. All these interconnected networks drive motor plasticity based on inputs received from somatosensory cortex.

Some of the same brain areas are known to be activated during a somatosensory training that does not involve active movement. For example, a recent fMRI study characterized the impact of four weeks of passive movement training of the wrist after stroke and found increased activation in the ventral premotor and parietal cortices of the contralesional hemisphere, while standard Bobath rehabilitation only increased ipsilesional activation and decreased contralesional activation^17^. There is additional evidence that both active and passive movements can drive motor learning and recovery, but active movements may be more beneficial because they induce enhanced neuronal activity in premotor cortex, SMA and somatosensory cortex when compared to passive movements^47^. Although the current study did not obtain neural activation data, evidence of the above studies delineates that the concurrent improvements in proprioceptive function and motor performance observed in the current study likely involve frontal motor cortical areas and parietal somatosensory areas.

### Summary and conclusion

The importance of proprioceptive signals for motor recovery and rehabilitation has long been recognized. Numerous behavioral treatment approaches have been introduced as a specialized proprioceptive training regimen designed to enhance proprioceptive function with the aim to improve or accelerate motor rehabilitation. The empirical evidence on the efficacy of these approaches has been mixed^12^, partly because the proprioceptive outcome measures reported were either not sensitive or because they infer improvements in proprioceptive function based on motor improvements. Here we employed sensitive measures of proprioceptive acuity and found that proprioceptive learning is linked to an improved motor performance in healthy adults.

## Methods

### Research Participants

Fourteen young healthy individuals (mean age: 27.29 yrs. ±4.63 yrs.) with no known neurological or musculoskeletal dysfunctions were recruited for participation. Handedness of the participants were determined by Edinburgh handedness inventory^48^. All participants were right-handed with a mean laterality index of 80.71 ± 13.42 on a scale of [−100 100] with −100 representing left-handedness and +100 for right-handedness. All participants provided informed consent for participation in the study. The study procedure and consent process was performed with approval and under the accordance of relevant guidelines and regulations by Institutional Review Board at the University of Minnesota.

### Wrist Robotic Device

**Figure 4:**
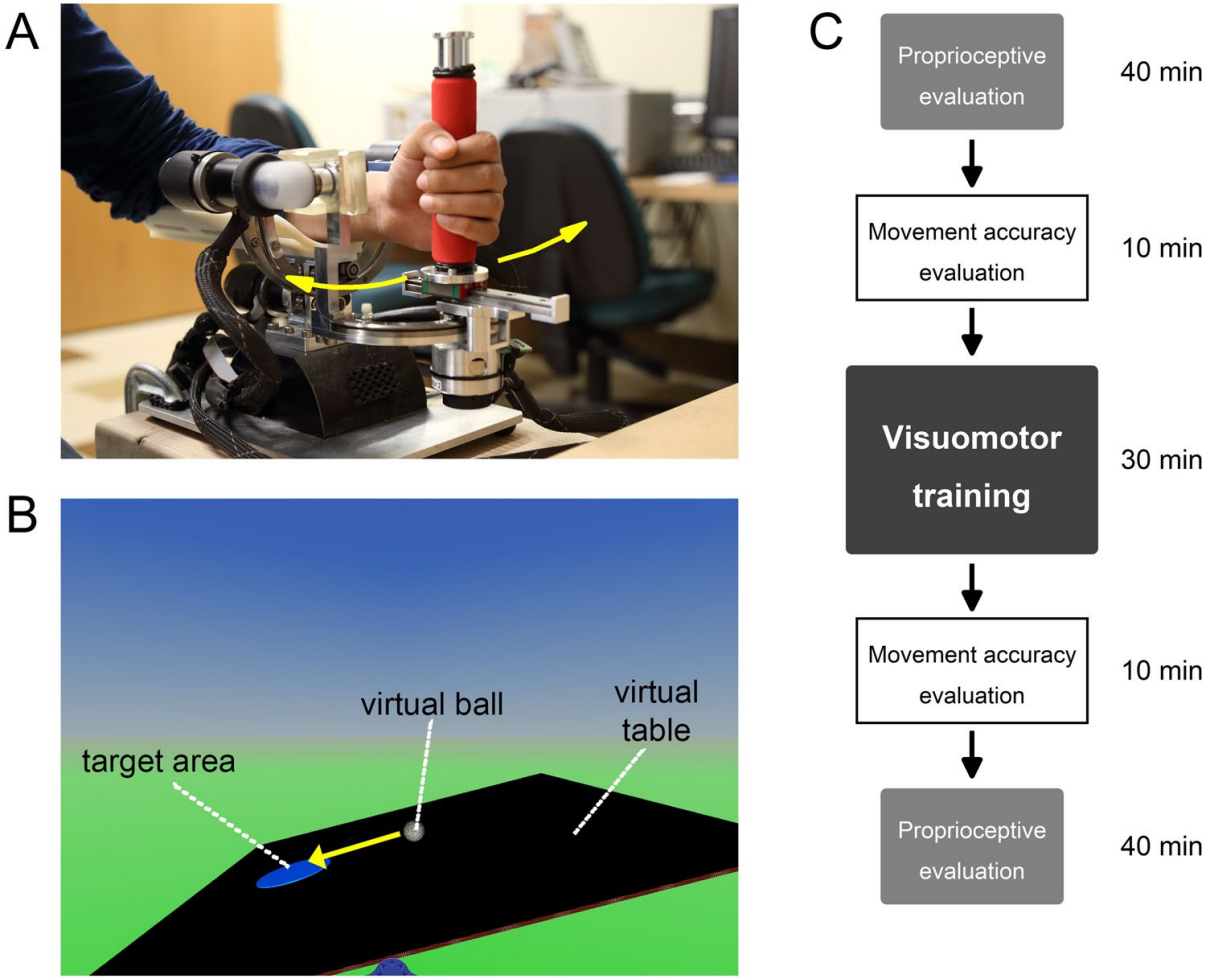
Haptic wrist robot. (**A**) A participant using the haptic wrist robot. The participant’s forearm is positioned on the wrist robot’s forearm support splint. This position is maintained during psychophysical evaluation, movement accuracy evaluation, and training task. (**B**) Monitor displaying virtual table and virtual ball during the training task. The subject extends the wrist causing the table in the virtual reality monitor to tip to the right resulting in displacement of the ball to the right. (**C**) Order of the experiment protocol. All participants were tested in the same order.

The wrist robot (Fig. 4A) is a three degree-of-freedom exoskeleton that allows for the full range of motion for the human wrist. It consists of a hand grip and a forearm support splint with Velcro straps to secure the participant’s forearm in a constant position. The haptic robot is a fully backdrivable system with the capability of delivering maximal torque comparable to the maximum torque levels of the human wrist. The device is powered by 4 brushless motors designed to provide an accurate haptic rendering and compensate for the weight and inertia of the device (i.e. the user does not perceive, nor needs to compensate the weight of the manipulator). It can deliver precise haptic, position and velocity stimuli at the wrist while being able to accurately encode the position of the wrist across time. Full technical details of the wrist robot are described elsewhere^36^. The robot was integrated with a virtual reality environment (VR) providing the user with a visual feedback of his/her wrist position during the execution of training task.

### Experimental Protocol

The experiment followed a single-group, one-treatment design with pre- and post-tests (see Fig. 4C). The proprioceptive acuity and motor accuracy assessments required different control modes of the robot, with required changes in its hardware and software settings, which took about 10 minutes each time. Baseline testing began with proprioceptive acuity assessment followed by motor accuracy assessment. During post-test, assessments were performed in the reverse order to minimize overall testing time. Both procedures are known to be reliable, but show no learning effects as people receive no performance feedback^31,36^. Thus, order of assessments was not randomized between participants. At the beginning of the experiment, all participants were briefed on the device and received practice trials to become familiar with the technology. This step assured that possible pre-posttest differences were not due to differences in device familiarity (i.e. familiarization effect).

### Visuomotor Wrist Training

Using a virtual reality environment in combination with the wrist robot, training required participants to use vision and proprioception to balance a virtual ball on a virtual table by making controlled, small amplitude wrist flexion/extension movements based on visual and haptic feedback received (Fig. 4B). During training, participants sat comfortably in a chair with their dominant forearm resting on the support splint of the wrist robot. When participants flexed or extended their wrist, the virtual table tipped either to the left or to the right respectively, resulting in the virtual ball moving as it would happen in real world. In each training trial, the goal for the participant was to move the ball to a circular target and to hold it there for 5 seconds. Within a training block (i.e. a level of task difficulty), a *balanced wrist position* of either 10°, 15° or 20° flexion from the neutral joint position corresponded to a fully balanced, horizontal position of the virtual board where the ball would not move. The level of difficulty increased by manipulating the virtual mass of the ball, the gain between wrist movement and the ball velocity, the dampening force on the ball velocity, and the gravity in the virtual environment. Advancing to the next level of difficulty, required the learner to increase the spatial and temporal accuracy of his/her wrist movements in order to be successful in moving the ball to the target. Within each level of difficulty, the participant first experienced the virtual board as horizontal at 10° flexion position. When the participant reached the virtual target within 45 s, a trial was scored to be successful and balanced wrist position changed to the next larger amplitude (either 15° or 20° flexion). If the trial was not successful, the target wrist position remained the same for the consecutive trial. Every participant completed a total of 45 training trials. That is, total number of training trials was the same for all participants, but the level of task difficulty they could achieve and experience, could vary between participants. Participants took about 30–40 minutes to complete the 45 training trials. If the participant was successful in every single trial, he/she would have experienced 15 levels of difficulty. Conversely, if the participant was unsuccessful in every single trial, he she would have stayed at level 1 at a 10° balanced wrist position. Participants could take a 2-minute break after every 15 trials.

### Evaluation of Sensory and Motor Function

#### Evaluation of Proprioceptive Acuity

**Figure 5:**
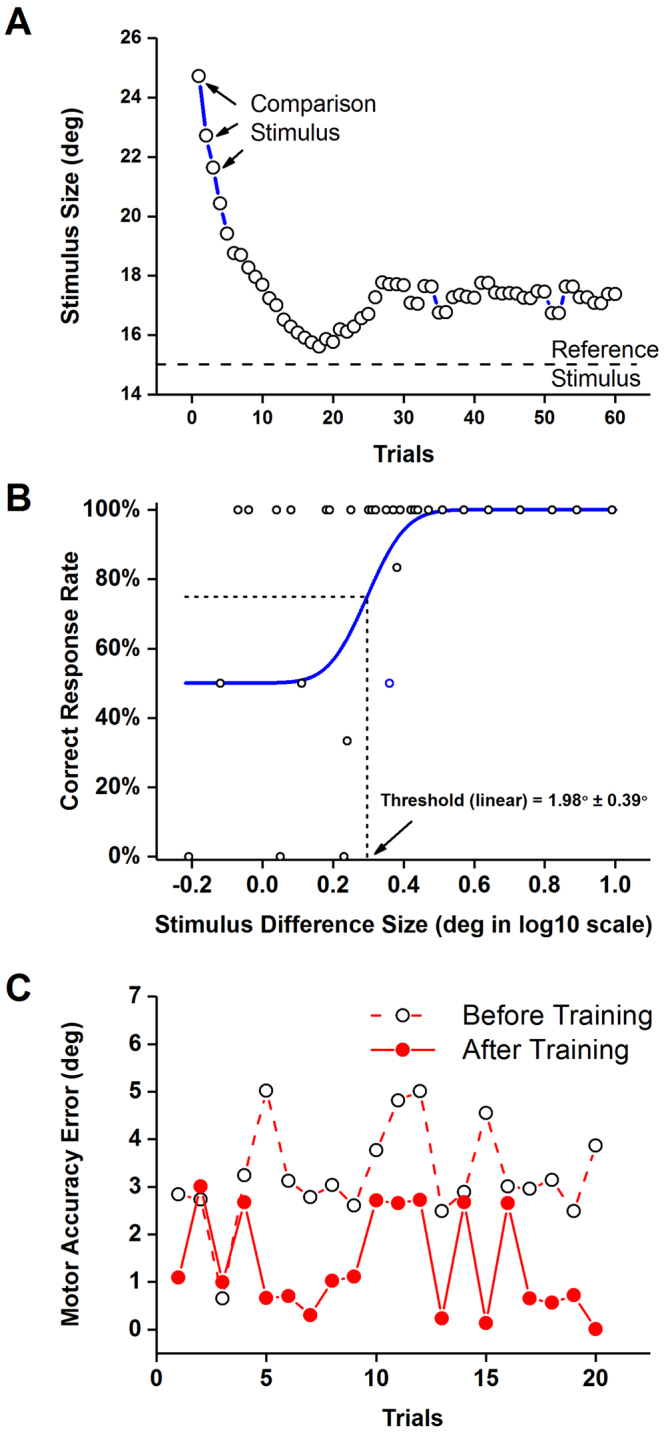
Assessment of proprioceptive acuity. (**A**) Changes in the stimulus size difference across trials of a single participant. Note how the trial-by-trial size difference was reduced quickly over the first 20 trials and probed values around the participant’s threshold. (**B**) The resulting proprioceptive acuity function based on participant’s verbal responses. The 75% correct response rate of the cumulative Gaussian distribution function refers to the proprioceptive discrimination threshold. (**C**) Movement accuracy evaluation. Exemplar data from one participant showing the error values across 20 trials before and after training.

Wrist position sense acuity was determined by a *psychophysical forced-choice paradigm* using the wrist robot. In each trial participants had to discriminate between two position stimuli (a standard position of 15° and a comparison position always greater than 15°). The robot passively moved the wrist at a velocity of 6°/s from neutral to either the standard or the comparison position. After experiencing both positions, participants had to verbally indicate which position was more flexed. The subsequent stimulus pairs of the next trial were determined based on the verbal response of the previous trial and the *stimulus difference size* between the standard and comparison using an adaptive Quest algorithm^49^. The Quest algorithm ensured that the stimulus difference size converged to the position sense discrimination thresholds almost monotonically (see Fig. 5A). The procedure was repeated for up to 80 trials depending on when convergence had been reached. Participants wore a pair of headphones playing pink noise and opaque glasses to eliminate auditory and visual cues. Participants took a 2-minute break after every 15 trials to avoid prolonged testing related recall bias and other cognitive biases. A participant’s verbal response for each trial was recorded along with the associated stimulus size difference. Position sense acuity was determined by fitting the response data using a cumulative Gaussian distribution function (see Fig. 5B). The stimulus size difference corresponding to 75% correct response rate was defined as the *proprioceptive discrimination threshold* (*DT*), representing the measure of position sense acuity.

#### Evaluation of Movement Accuracy

The spatial accuracy of wrist movement was evaluated in a novel goal-directed wrist pointing task. One differentiates two aspects of accuracy: bias and precision^50^. Here, *bias* indicates how close a wrist joint position corresponds to the desired target position and *precision* represents the random error between independent repeated responses. The transfer task to evaluate movement accuracy was different from the training task. During training participants performed continuous, small-amplitude corrective movements in the presence of vision. In contrast, spatial movement accuracy was assessed in a task that was not part of the training and required discrete, ramp-like wrist flexion movements to a relative large target amplitude in the absence of vision. In this task, the wrist robot first passively moved the wrist to a target position of 15° flexion from the neutral joint position, held the position for 2 seconds and moved the wrist back to the neutral position. The participant then actively moved the wrist to the target position. The procedure was repeated for 20 trials (see Fig. 5C). Similar to the position sense discrimination testing, participants wore headphones and opaque glasses, that is, they had to rely on proprioceptive feedback to experience and then reproduce the target position. For each trial, the difference between target and achieved wrist position was calculated. Subsequently, the mean of these difference values was computed for each participant representing a *movement accuracy error* (*MAE*).

### Data Analysis and Measurements

All participants were evaluated for position sense and movement accuracy before and after training to determine the influence of proprioceptive training (Fig. 1). The respective proprioceptive discrimination threshold and movement accuracy error were tested for normality using the Kolmogorov-Smirnov test. Test statistics showed that both dependent variables, proprioceptive discrimination thresholds and movement accuracy error were normally distributed. Thus, parametric statistical analyses were performed on both variables. Separate one-way repeated measures Analysis of Variance procedures (ANOVA) were performed on discrimination threshold and movement accuracy error to identify the effects of training on proprioceptive function and movement accuracy. Absolute and relative improvements in the proprioceptive discrimination threshold and movement accuracy error before and after training were computed to better understand the effects of training on these measures. Absolute improvements were calculated by computing the absolute differences between pre-test and post-test measurements, whereas relative improvements are calculated by dividing the absolute differences between pre-and post-test measurements by pre-test measurements. A custom-written algorithm based on R: A language and environment for statistical computing^51^ was used for statistical analyses.

### Data availability

Datasets generated during the current study are available from the corresponding author on reasonable request.
